# DNAJB6: A guardian against neurodegeneration

**DOI:** 10.4103/NRR.NRR-D-24-01504

**Published:** 2025-06-19

**Authors:** Jónvá Hentze, Anna Gelman, Tomasz Brudek, Christian Hansen

**Affiliations:** 1 Department of Nutrition, Exercise and Sports, Faculty of Science, University of Copenhagen, Copenhagen, Denmark; 2 Department of Technology, University College Copenhagen, Copenhagen, Denmark; 3 Research Laboratory for Stereology and Neuroscience, Bispebjerg-Frederiksberg Hospital, University Hospital of Copenhagen, Copenhagen, Denmark

**Keywords:** aggregation, chaperones, clinical tissues, DNAJB6, human brain, neurodegeneration, neurodegenerative diseases, therapeutic target

## Abstract

Amyloid protein aggregation plays a major role in multiple neurodegenerative diseases and is likely the primary driving force for the progression of most of these diseases. Multiple recent studies have highlighted that the DNAJ homolog subfamily B member 6 (DNAJB6) chaperone is particularly interesting, when it comes to preventing amyloidogenic proteins from aggregating. It has been shown that DNAJB6 can prevent the aggregation of polyglutamine-expanded proteins in models of Huntington’s disease. Likewise, it can suppress aggregation of α-synuclein in models of Parkinson’s disease and other synucleinopathies. Finally, it has been shown that DNAJB6 can block aggregation of multiple additional amyloid proteins involved in Alzheimer’s disease and other tauopathies as well. We believe there is yet much to learn about the protective role of DNAJB6 in the brain, but this focused review summarizes, what we know so far of this chaperone. It describes the biological role of DNAJB6 in the brain and its interaction with Hsp70, with particular emphasis on the studies that show its ability to prevent amyloid protein aggregation *in vitro* and *in vivo*. Moreover, recent work on dysregulation of the expression of DNAJB6 in brain clinical tissue is discussed. Finally, we discuss potential therapeutic perspectives as we believe this protein is a promising druggable target.

## Introduction

Protein homeostasis is crucial for the survival of all living cells. To ensure proper protein folding, cells must be able to identify numerous different structures and assess whether they require folding or refolding. Molecular chaperones play a key role in protein homeostasis, working in coordination with other cellular quality control systems. They are highly conserved, abundant proteins, ubiquitously expressed across different cellular compartments and tissues (Hageman et al., 2011; Jayaraj et al., 2020; Shemesh et al., 2021). Chaperones assist in maintaining protein homeostasis by facilitating processes such as translocation, refolding, and degradation. This also includes preventing the aggregation of unfolded or misfolded proteins (Nillegoda et al., 2018; Prodromou et al., 2023).

Expression of some chaperones can increase in response to cellular stress, such as an imbalance in antioxidants and free radicals, exposure to toxins, and change in temperature (Nillegoda et al., 2018). The main cytosolic response to stress is the “heat shock response,” which is characterized by increased expression of multiple chaperones. These chaperones are named “heat shock proteins” (HSPs), as they were first identified as proteins induced by heat shock (Schlesinger, 1990). However, it is important to note that far from all of them are heat-shock inducible. Deficiencies in one or more factors of the protein homeostasis system might lead to proteotoxic stress, such as the accumulation of toxic amyloidogenic protein species, ultimately resulting in cell death. The clearance of toxic proteins is critical for all cells, but is especially important in post-mitotic cells, such as neurons, since they are not readily replaced (Di Domenico and Lanzillotta, 2022). In neurons, maintenance of protein homeostasis is essential for healthy aging, as failure or modulation of protein homeostasis and the heat shock response, is a likely cause of age-related neurodegenerative protein-misfolding diseases known as proteinopathies (Di Domenico and Lanzillotta, 2022).

DNAJ homolog subfamily B member 6 (DNAJB6) is a J-domain protein (JDP) recognized for its protective effects in the brain. This review synthesizes findings from various research studies, including clinical studies, which elucidate its role in these conditions, with particular emphasis on its involvement in protein aggregation and neurodegeneration. While other JDPs also contribute to neuroprotection, their roles have been comprehensively covered in other reviews (Kampinga and Craig, 2010; Kampinga and Bergink, 2016; Hasegawa et al., 2017; Zhang et al., 2023). Here, we briefly introduce the JDP family of chaperones before focusing on DNAJB6. Importantly, understanding how DNAJB6 can prevent the formation and propagation of amyloidogenic seeds, may pave the way for the design and development of future therapeutic strategies.

## Search Strategy

Studies cited in this narrative review were published between 1963 and 2025. Studies published in the recent years were prioritized. Studies were searched on the PubMed database using terms such as: DNAJB6, neurodegeneration, aggregation, heat shock proteins, J-domain, human brain, DNAJ, expression, Huntington’s disease, Parkinson’s disease, a-synuclein.

## The J-Domain Family of Proteins

**Figure 1:**
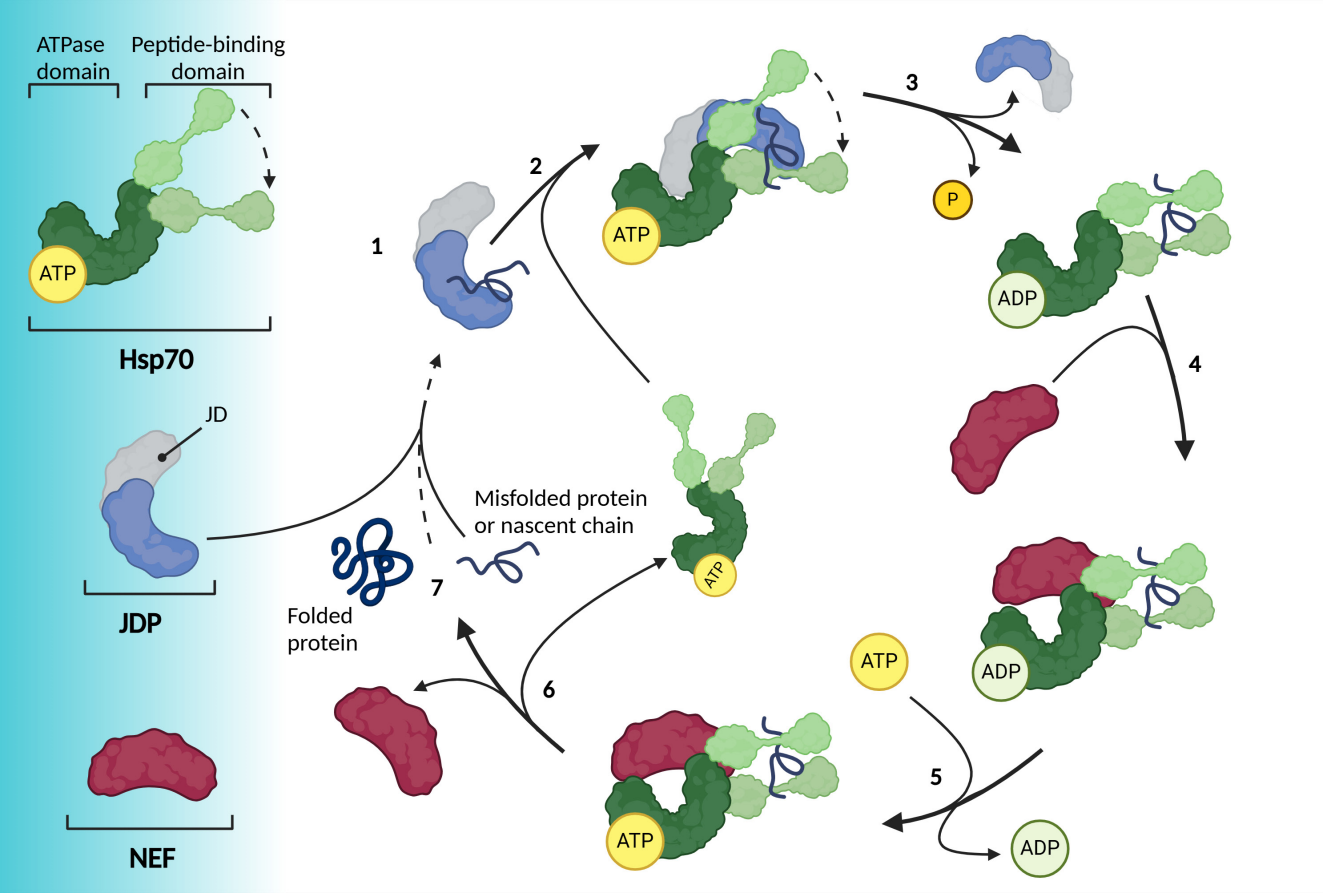
Hsp70 machinery cycle of protein folding. The role of the JDPs in the Hsp70 machinery: (1) JDPs bind to the client protein, which can be a nascent chain or a misfolded protein. (2) JDP interacts with the Hsp70 ATPase domain via its JD. (3) The client protein binds to the open peptide-binding domain of Hsp70. While ATP hydrolysis is activated by the JD and the client protein, it causes a conformational change in the peptide-binding domain of Hsp70, “closing” it, and thus stabilizing the interaction with the client protein. (4) NEF has a high affinity for Hsp70-ADP and thus binds to Hsp70. (5) After a conformational change to the ATPase domain, ADP dissociates, and ATP can bind to Hsp70 again. (6) The client protein has a lower affinity for Hsp70-ATP, and thus dissociates together with NEF. (7) The client protein has reached its native conformation. If the client protein does not reach its folded or native state, the cycle starts over. Created with BioRender.com. ADP: Adenosine diphosphate; ATP: adenosine triphosphate; Hsp70: heat shock protein 70; JD: J-domain; JDP: J-domain protein; NEF: nucleotide exchange factor.

The challenge of assuring proper folding of a diverse proteome is resolved through the coordinated action of molecular chaperones with their co-factors, known as co-chaperones. Co-chaperones from the family of JDPs play a crucial role in preventing the development of several proteinopathies, by suppressing protein aggregation and cytotoxicity (Hageman et al., 2010; Rose et al., 2011; Hasegawa et al., 2017; Marszalek et al., 2024). JDPs bind to misfolded or unfolded proteins and modulate folding through recruitment to and interactions with proteins of the Hsp70 family (Kampinga and Craig, 2010). In addition to their interaction with JDPs, the Hsp70 machinery interacts with nucleotide exchange factors. Specifically, JDPs deliver unfolded or misfolded client proteins to Hsp70 and bind to its ATP’ase domain, modulating its ATP’ase activity, while nucleotide exchange factors facilitate the exchange of ADP for ATP (Vos et al., 2008; Johnson et al., 2022), ultimately resulting in the release of the properly folded client protein (**Figure 1**).

Bioinformatic analysis by Hageman and Kampinga (2009) suggests that while most JDP members are cytosolic proteins, some are localized to the endoplasmic reticulum, mitochondria, or nucleus. Additionally, JDPs outnumber Hsp70 in various cellular compartments, which suggests that a single Hsp70 can interact with multiple JDP partners (Hageman and Kampinga, 2009). It has also been proposed that JDPs may have biological roles independent of their interaction with the Hsp70 machinery (Hageman et al., 2010; Gillis et al., 2013; Banerjee et al., 2022), but this topic is beyond the scope of this review.

There are 50 different members of the human JDP family ranging in size from approximately 12 to 521 kDa (Kampinga et al., 2009). The range in sizes reflects the functional variance among the members, which may also be due to their varied domain structures (Faust et al., 2020). Despite this, all JDPs share the defining feature of a conserved J-domain (JD) (Kampinga et al., 2009). The JD is a 70 aa domain, consisting of four α-helices and a loop through which it binds the ATPase domain of Hsp70s. A key element within this domain is the highly conserved histidine-proline-aspartic acid (HPD) motif located in a loop between two of the α-helices, critical for binding to Hsp70s (Kampinga and Craig, 2010). Given the strong primary sequence homology between all JDs across different proteins and species, the core functionality of the JD, particularly the HPD motif, appears to be universally conserved, ensuring a consistent mechanism of interaction with Hsp70.

### J-domain protein subfamilies

**Figure 2:**
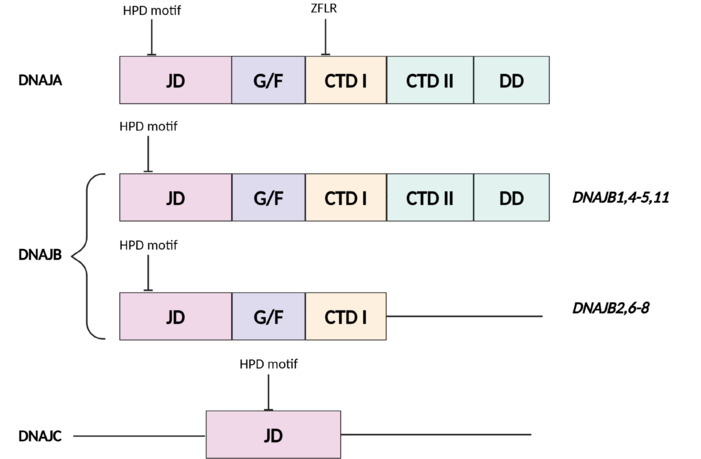
The JDP subfamilies. General structural representation of the three different JDP subfamilies: DNAJA proteins consist of an N-terminal JD, containing the HPD motif, followed by a G/F and a ZF and a variable CTD. DNAJB proteins are in general similar to DNAJA proteins but lack the ZF, and can be divided into two subgroups depending on domain/region architecture. DNAJC proteins are variable but contain a JD that can be positioned anywhere in the protein. Created with BioRender.com. CTD: C-terminal domain; DNAJA: DNAJ homolog subfamily A; DNAJB: DNAJ homolog subfamily B; DNAJC: DNAJ homolog subfamily C; G/F: glycine-phenylalanine-rich region; HPD: histidine-proline-aspartic acid; JD: J-domain; JDP: J-domain protein; ZF: zinc-finger domain.

The JDP family is classified into three subfamilies based on homology: DNAJA, DNAJB, and DNAJC (**Figure 2**). These subfamilies display considerable structural and sequence divergence outside of the JD (Kampinga and Craig, 2010), reflecting their involvement in a wide range of cellular functions across various tissues.

DNAJA members are highly homologous and represent the closest eukaryotic orthologs of the canonical JDP in *E. coli* (Vos et al., 2008). DNAJAs contain a highly conserved architecture consisting of an N-terminal JD, a glycine/phenylalanine-rich (G/F) region, a cysteine-rich region with a zinc-finger motif, and a variable C-terminal extension. The C-terminal contains a β-stranded C-terminal domain containing a zinc-finger region and a dimerization domain. DNAJB proteins are similar to DNAJA but lack the zinc-finger motif. Further, the DNAJB subfamily can be divided into two subgroups, one of which lacks the dimerization domain, while DNAJC proteins only have in common that they contain the JD, which can be positioned at various locations within the protein (Kampinga and Craig, 2010; **Figure 2**). Compared to DNAJA and DNAJB subfamilies, the DNAJC subfamily is significantly larger and more diverse. DNAJC proteins frequently perform specialized roles within different cellular compartments, for instance facilitating mitochondrial import and participating in endoplasmic reticulum-associated degradation (Melnyk et al., 2023; Jiahui et al., 2025). The diversity is due to the presence of unique domains and/or regions that allow DNAJCs to function in specific signaling pathways, degradation, and subcellular trafficking (van Huizen et al., 2003; Hirst et al., 2008; Melnyk et al., 2023). However, the DNAJC subfamily is far more diverse than **Figure 2** exemplifies, exhibiting a wider range of domain and region architecture than represented here. For simplicity, only the JD is shown, as the diversity among DNAJC proteins has been extensively described elsewhere (Kampinga and Craig, 2010). The classification is based on historical classification and does not necessarily reflect the biochemical functions or mechanisms of action of the group members within the specific subfamilies (Kampinga and Craig, 2010). Of the three subfamilies the DNAJB subfamily has been most extensively studied and consists of 14 members: DNAJB1-DNAJB14 (Kampinga et al., 2009). One of the most well-documented JD proteins regarding human protein folding/degradation is DNAJB1 (Gao et al., 2015; Alharbi et al., 2023), which has also been found to be the most abundant JDP transcript after heat shock (Hageman et al., 2011), indicating its involvement in proteotoxic stress. A neighbor-joining phylogeny analysis by Hageman et al. (2011) pointed to the existence of two groups within the DNAJB subfamily. The first group showed homology to DNAJB1, DNAJB4, and DNAJB5, while the second group consisted of DNAJB2 and DNAJB6-8 (Hageman et al., 2011). Members of the second group share a high degree of sequence homology at the CTD, which is different from other comparable domains. Interestingly, the second group (DNAJB2, DNAJB6, and DNAJB8 specifically), have been identified as potent inhibitors of aggregation of amyloidogenic proteins (Hageman et al., 2010; Gillis et al., 2013). DNAJB8 transcripts have only been detected in testis, while DNAJB6 is expressed ubiquitously (Hageman et al., 2010), but has a higher expression in some tissues, such as brain tissue (Seki et al., 1999; Chuang et al., 2002).

## Expression of the Two DNAJB6 Isoforms in Cells and Tissues

**Figure 3:**
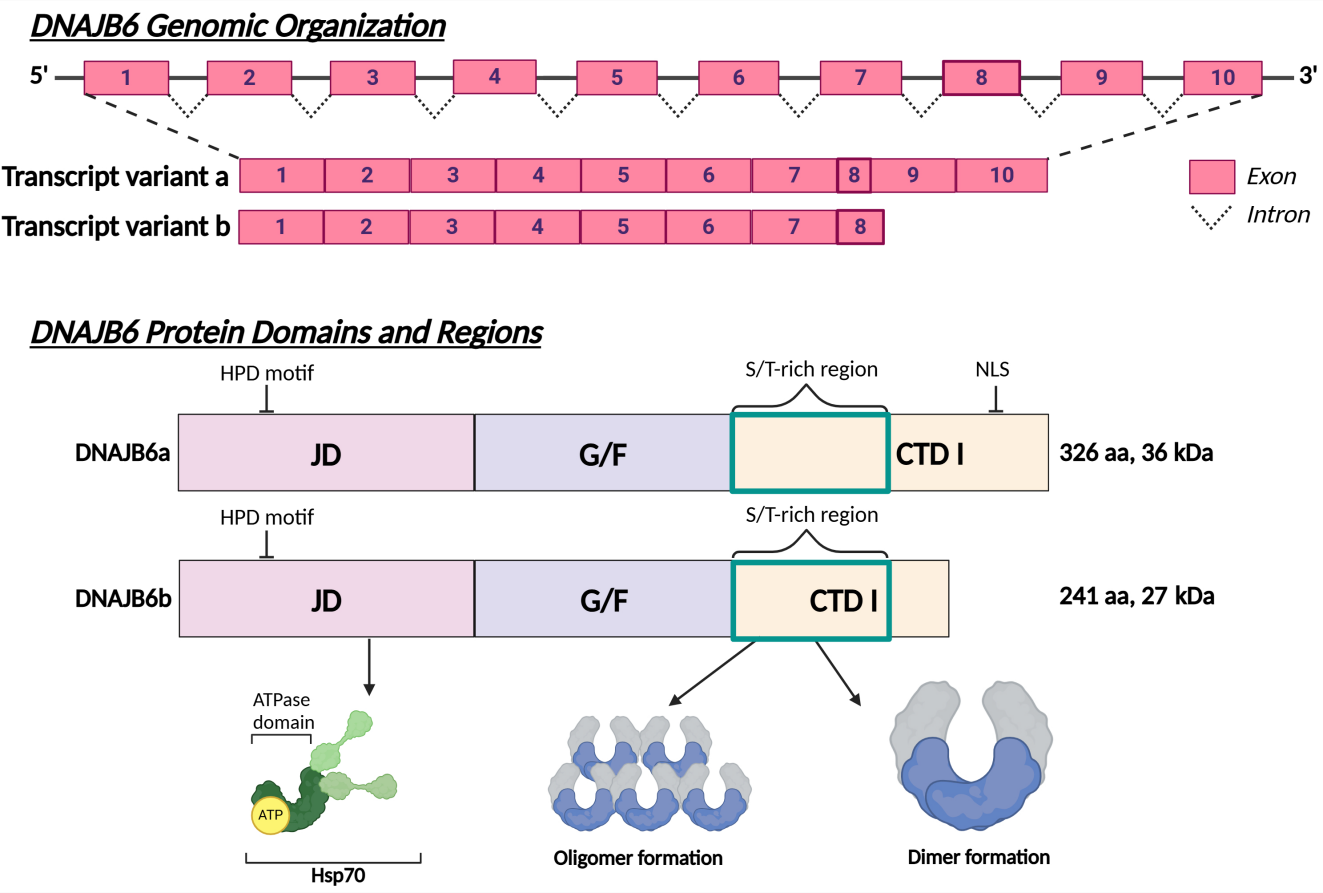
The DNAJB6 isoforms. Structural representation of the DNAJB6 isoforms: (A) Map of the *DNAJB6* gene structure. Alternative splicing of the *DNAJB6* gene generates two isoforms, DNAJB6a and DNAJB6b, which differ at their 3’ end. Both isoforms share exons 1–7 and a portion of exon 8. In DNAJB6a, exon 8 is shorter and spliced to exons 9 and 10. In contrast, DNAJB6b features a longer exon 8, which includes a sequence encoding 10 amino acids unique to the DNAJB6b isoform. (B) Linear illustration of DNAJB6a and DNAJB6b on protein level. The protein is illustrated with the various domains and their involvement in different cellular processes. The alternatively spliced CTD is indicated. The length of DNAJB6a and DNAJB6b is 326 and 241 amino acids, respectively. Created with BioRender.com. ATP: Adenosine triphosphate; CTD: C-terminal domain; DNAJB: DNAJ homolog subfamily B; G/F: glycine/phenylalanine-rich region; HPD: histidine-proline-aspartic acid tripeptide; Hsp70: heat shock protein 70; JD: J-domain; NLS: nuclear localization signal; S/T: serine/threonine-rich region.

The JD co-chaperone DNAJ homolog subfamily B member 6 (DNAJB6), previously known as the *mammalian relative of DnaJ* (Seki et al., 1999), belongs to the DNAJB subfamily of proteins. DNAJB6 exists as two alternatively spliced isoforms: DNAJB6a (36 kDa, 326 aa) and DNAJB6b (27 kDa, 241 aa) (Hanai and Mashima, 2003; **Figure 3**). Both isoforms share the first seven exons, while DNAJB6a has shorter exon 8, which is spliced to the last two exons. In contrast, DNAJB6b has a longer exon 8 that includes a sequence encoding 10 amino acids unique to the DNAJB6b isoform (**Figure 3**). On the protein level, both isoforms share the N-terminal JD, the G/F-rich region, and the serine/threonine (S/T)-rich region, which is involved in interactions with client proteins (Kakkar et al., 2016b). However, the CTDs are unique to each isoform. DNAJB6a contains a nuclear localization signal within its CTD, leading to its primary localization in the nucleus (Hanai and Mashima, 2003; Mitra et al., 2008), though it is also found in the nuclear envelope (Ding et al., 2016). The shorter DNAJB6b isoform is primarily cytosolic but can also be found in the nucleus (Hanai and Mashima, 2003), where it has been shown to accumulate during cellular stress, such as heat shock (Dai et al., 2005; Andrews et al., 2012) and hypoxia (Andrews et al., 2012). Recently, it has also been shown to be enriched in the nuclear envelope, and to play a prominent role in the biogenesis of nuclear pore complexes (NPCs) (Kuiper et al., 2022). During NPC biogenesis, phenylalanine-glycine nucleoporins are prone to aggregation due to their intrinsically disordered nature, which can disrupt proper NPC assembly. Notably, the S/T-rich region of DNAJB6 has been shown to interact with the FG-rich regions of phenylalanine-glycine nucleoporins, thereby preventing unwanted aggregation (Kuiper et al., 2022).

The two isoforms of the *DNAJB6* gene are ubiquitously expressed in various human and mouse tissues (Hunter et al., 1999; Seki et al., 1999; Chuang et al., 2002; Hageman et al., 2010) with *DNAJB6b* being expressed at a higher level than *DNAJB6a* in both tissues and cell lines (Hageman et al., 2010; Arkan et al., 2021; Folke et al., 2021). Within the brain, higher levels of *DNAJB6b* are found in the hippocampus and thalamus, compared to the amygdala, *substantia nigra* (SN), corpus callosum, and caudate nucleus (Chuang et al., 2002). Consistent with this, high amounts of DNAJB6 protein have been found in the brain in rats (Chuang et al., 2002), but a decrease is seen in the striatum in a mouse model of Parkinson’s disease (PD) (Choi et al., 2011).

The significant expression of DNAJB6 in the brain, suggests a critical role in maintaining neuronal health and stability, as well as being an important factor in counteracting protein misfolding and aggregation – processes that are detrimental in neurodegenerative diseases. In several models of neurodegeneration, DNAJB6 has shown a remarkable ability to suppress the aggregation of various amyloid proteins, such as huntingtin (Htt), tau, and α-synuclein, highlighting its protective potential against toxic aggregates of the brain (Hageman et al., 2010; Mansson et al., 2014a; Deshayes et al., 2019; Chang et al., 2023).

## DNAJB6 Suppresses Amyloid Protein Aggregation in Models of Neurodegenerative Diseases

The ability of DNAJB6 to impair amyloidogenic protein aggregation has been explored in multiple experimental models (**Table 1**), and accumulating evidence suggests that therapies targeting DNAJB6 could be beneficial across multiple neurodegenerative disorders. Most well studied is the ability of DNAJB6 to reduce the aggregation of polyglutamine (polyQ) proteins in Huntington’s disease (HD) models. HD is a genetically inherited disease that is caused by a CAG expansion in the *Huntingtin* gene, resulting in an extended polyQ stretch at the N-terminus of the Htt protein (Saade and Mestre, 2024). The onset of HD is inversely related to the length of the polyQ expansion, with longer expansions leading to earlier disease onset (Kuiper et al., 2017).

**Table 1:** DNAJB6 suppresses aggregation of several amyloidogenic proteins *in vitro* and *in vivo*

Disease	Amyloidogenic protein	Animal model	Cellular model	*In vitro* model	DNAJB6 overexpression	DNAJB6 KD/KO	Aggregation impairment	Toxicity protection	Disease pathology protection
HD	*HttExon1Q45*			Mansson et al., 2014a; Kakkar et al., 2016b			Mansson et al., 2014a; Kakkar et al., 2016b		
	*HttExon1Q51*		Lee et al., 2020		Lee et al., 2020		Lee et al., 2020		
	*HttExon1Q71*		Thiruvalluvan et al., 2020		Thiruvalluvan et al., 2020	Thiruvalluvan et al., 2020	Thiruvalluvan et al., 2020		
	*HttExon1Q74*		Hageman et al., 2010, 2011; Kakkar et al., 2016b; Rodriguez-Gonzalez et al., 2020; Joshi et al., 2021		Hageman et al., 2010, 2011; Kakkar et al., 2016b; Rodriguez-Gonzalez et al., 2020; Joshi et al., 2021	Rodriguez-Gonzalez et al., 2020; Joshi et al., 2021	Hageman et al., 2010, 2011; Kakkar et al., 2016b; Rodriguez-Gonzalez et al., 2020; Joshi et al., 2021	Rodriguez-Gonzalez et al., 2020	
	*HttExon1-12Q100*	Bason et al., 2019			Bason et al., 2019		Bason et al., 2019	Bason et al., 2019	
	*HttExon1Q103*		Gillis et al., 2013		Gillis et al., 2013	Gillis et al., 2013	Gillis et al., 2013		
	*HttExon1Q119*		Hageman et al., 2010; Kakkar et al., 2016b		Hageman et al., 2010; Kakkar et al., 2016b		Hageman et al., 2010; Kakkar et al., 2016b	Hageman et al., 2010	
	*HttExon1Q150*		Chuang et al., 2002		Chuang et al., 2002		Chuang et al., 2002	Chuang et al., 2002	
	*HttExon1Q201*	Kakkar et al., 2016b; Joshi et al., 2021			Kakkar et al., 2016b; Joshi et al., 2021		Kakkar et al., 2016b; Joshi et al., 2021		Kakkar et al., 2016b; Joshi et al., 2021
PD	*Parkin (C289G)†*		Rose et al., 2011; Kakkar et al., 2016a		Rose et al., 2011; Kakkar et al., 2016a		Rose et al., 2011; Kakkar et al., 2016a		
	*α-Synuclein*	Arkan et al., 2021	Aprile et al., 2017; Deshayes et al., 2019; Arkan et al., 2021		Aprile et al., 2017; Arkan et al., 2021	Aprile et al., 2017; Deshayes et al., 2019	Aprile et al., 2017; Deshayes et al., 2019; Arkan et al., 2021	Arkan et al., 2021	Arkan et al., 2021
AD	*Aβ42*		Hussein et al., 2015	Mansson et al., 2014b, 2018; Osterlund et al., 2023; Carlsson et al., 2024	Hussein et al., 2015		Mansson et al., 2014b, 2018; Osterlund et al., 2023; Carlsson et al., 2024		
	*Tau (P301L)†*	Chang et al., 2023	Chang et al., 2023		Chang et al., 2023	Chang et al., 2023	Chang et al., 2023		Chang et al., 2023
ALS	*TDP-43*		Udan-Johns et al., 2014		Udan-Johns et al., 2014	Udan-Johns et al., 2014	Udan-Johns et al., 2014		

†: Mutation at this site. Aβ: Amyloid-beta; AD: Alzheimer&s disease; ALS: amyotrophic lateral sclerosis; DNAJB: DNAJ homolog subfamily B; HD: Huntington’s disease; KD: knockdown; KO: knockout; PD: Parkinson’s disease; TDP-43: TAR DNA binding protein 43.

An initial study by Chuang et al. (2002) demonstrated that DNAJB6b can inhibit Htt aggregation and reduce cellular toxicity in a cell model of HD, though it had little or no effect on clearing pre-existing aggregates. However, extensive research by the Kampinga lab has linked DNAJB6 to the inhibition of polyQ-expanded Htt aggregation. They have demonstrated that DNAJB6b effectively suppresses polyQ aggregation without disaggregating existing aggregates (Hageman et al., 2010). In addition, it has been demonstrated that DNAJB6b inhibits aggregate formation at remarkably low sub-stoichiometric molar ratios of chaperone to peptide (Mansson et al., 2014a). This suggests that DNAJB6b interacts with oligomeric/aggregated polyQ species rather than monomeric forms (Kakkar et al., 2016b). Additionally, DNAJB6b has been detected in the core of polyQ aggregates, whereas Hsp70 is localized primarily at the periphery, which supports the idea that DNAJB6b engages polyQ peptides early in the aggregation process, in contrast to Hsp70 (Gillis et al., 2013). Furthermore, DNAJB6b has been shown to mechanistically inhibit polyQ aggregation independently of Hsp70 (Hageman et al., 2010; Gillis et al., 2013; Mansson et al., 2014a), although a dependency is required for full activity in cells (Kakkar et al., 2016b). However, when proteasomal degradation is blocked or Hsp70 is inhibited, the protective effect of DNAJB6b on aggregation is abolished, indicating that DNAJB6b cooperates with Hsp70 to facilitate the degradation of misfolded proteins (Hageman et al., 2010; Kakkar et al., 2016b). Furthermore, it has been observed in multiple cellular studies that DNAJB6b protects against polyQ aggregation and toxicity, using either DNAJB6 overexpression (Hageman et al., 2010, 2011; Gillis et al., 2013) or DNAJB6 knockout (KO) studies (Rodriguez-Gonzalez et al., 2020). Importantly, in a mouse model of HD, Kakkar et al. (2016b) demonstrated that overexpression of DNAJB6 in the brain resulted in delayed polyQ aggregation and prolonged lifespan of the mice.

Additionally, the expression of human DNAJB6 in neurons has been found to provide both cell-autonomous and non-cell-autonomous protection against polyQ-mediated neurodegeneration in *Drosophila* (Fayazi et al., 2006; Bason et al., 2019). In the study by Bason et al. (2019), a significant proportion of DNAJB6 expressing astrocytes contained neuronal-derived polyQ aggregates, suggesting that astrocytes may take up these aggregates from neurons, thereby contributing to neuroprotection. This suggests that DNAJB6 plays a role in glial cells and in mitigating neuronal degeneration.

A recent study using patient-derived induced pluripotent stem cells compared polyQ aggregation during neuronal differentiation (Thiruvalluvan et al., 2020). In cells derived from an HD patient, glutamate treatment led to aggregate formation in neurons, but not in neuronal progenitors. A marked reorganization of the chaperone network during differentiation was observed, with reduced DNAJB6 expression in neurons compared to neural stem cells. Re-expression of DNAJB6 in neurons reduced aggregation, while knockdown of DNAJB6 in progenitors promoted aggregation (Thiruvalluvan et al., 2020). This underscores the importance of DNAJB6 in preventing polyQ aggregation during neurodevelopment. Overexpression of DNAJB6b in cells has been shown to influence protein folding in co-cultured cells without direct contact, an effect mediated through the transfer of DNAJB6b encapsulated within extracellular vesicles (EVs) (Joshi et al., 2021). The ability of DNAJB6b to be packaged into EVs, which are endocytosed into recipient cells, represents a novel mechanism in which DNAJB6b can prevent misfolding in other cells than those where it is originally expressed, potentially mitigating the effects of protein misfolding and aggregation in these recipient cells. Indeed, neural stem cell-derived EVs loaded with DNAJB6b have been demonstrated to suppress polyQ aggregation in both cellular and mouse models of HD (Joshi et al., 2022).

Beyond its protective role against polyQ aggregation in HD, DNAJB6 has also been shown to inhibit the aggregation of α-synuclein, a key protein in PD pathology. A hallmark of PD pathology is the accumulation of Lewy bodies in the cytoplasm of neurons, with aggregated α-synuclein as the most abundant protein (Spillantini et al., 1997). Both *in vitro* and *in vivo* studies have shown that Hsp70 can prevent aggregation of α-synuclein (Auluck et al., 2002; Aprile et al., 2015; Gao et al., 2015). In line with this, Aprile et al. (2017) showed *in vitro* that DNAJB6 impairs the aggregation of α-synuclein in an Hsp70-dependent manner. Furthermore, increased α-synuclein aggregation was observed in DNAJB6 KO cells, which could be abolished by re-introducing DNAJB6 in the cells (Aprile et al., 2017). Subsequently, it was demonstrated that DNAJB6 also impairs α-synuclein preformed fibril seeded α-synuclein aggregation and that it targets α-synuclein degradation via the proteasome (Deshayes et al., 2019), using a fluorescence resonance energy transfer based cellular system to detect α-synuclein, originally designed by Marc Diamond and co-workers (Holmes et al., 2014). In a rat model of PD, induced by overexpressing α-synuclein in the brain, co-expression of DNAJB6b diminished α-synuclein induced neuronal cell death in the SN and prevented motor impairments of the rats, demonstrating that DNAJB6 protects against the development of PD, in an animal model of the disease (Arkan et al., 2021). In summary, there is substantial experimental evidence to support the importance of DNAJB6 in preventing α-synuclein from aggregating, as this has been shown *in vitro*, in cellular models and animal models.

The majority of the studies on the role of DNAJB6 in preventing amyloid protein aggregation have been conducted on the DNAJB6b isoform, whereas research on the DNAJB6a isoform, in this regard, remains sparse. However, a study by Gillis et al. (2013) has shown that DNAJB6a overexpression can lead to the inhibition of polyQ aggregation in cellular models. In contrast, specific KO of the DNAJB6a isoform in cells did not lead to increased α-synuclein aggregation, whereas KO of both isoforms did, suggesting that the DNAJB6a may not be important for inhibition of α-synuclein aggregation (Aprile et al., 2017). However, while α-synuclein is primarily localized in the cytoplasm, whereas polyQ primarily is localized in the nucleus, both isoforms may be important for inhibiting amyloid protein aggregation, potentially playing distinct roles in different cellular compartments.

In addition to the aforementioned diseases, DNAJB6 has been implicated in amyotrophic lateral sclerosis, a neurodegenerative disorder characterized by the aggregation of proteins such as TAR DNA binding protein 43 (TDP-43) and Fused in sarcoma. DNAJB6 has been shown to interact with these misfolded proteins, influencing their aggregation and toxicity (Kinger et al., 2023). For example, DNAJB6 may interact with the CTD of TDP-43, maintaining its solubility and preventing its aggregation similar to its effect on polyQ aggregates (Udan-Johns et al., 2014). This interaction underscores the role of DNAJB6 in modulating protein homeostasis imbalances often disrupted in amyotrophic lateral sclerosis, potentially slowing disease progression (Udan-Johns et al., 2014; Kinger et al., 2023). In models of Alzheimer’s disease (AD), it has been suggested that DNAJB6 may be a modulator of amyloid-beta (Aβ) aggregation. DNAJB6 can bind to Aβ peptides *in vitro*, preventing their aggregation into toxic fibrils, which are characteristics of AD pathology (Mansson et al., 2018). Similar to its action on polyQ proteins, DNAJB6 can maintain Aβ42 peptide solubility, keeping it available for degradation (Mansson et al., 2018). It inhibits aggregate formation at remarkably low sub-stoichiometric molar ratios of chaperone to peptide (Mansson et al., 2014b) suggesting that, as with polyQ peptides, DNAJB6b interacts with oligomeric/aggregated Aβ rather than monomeric (Mansson et al., 2014b, 2018; Osterlund et al., 2020). However, it should be noted that studies performed so far, have been conducted primarily *in vitro* with recombinant proteins, and future experiments using cellular and animal models, will highlight the potential of DNAJB6 in inhibiting Aβ aggregation.

Furthermore, DNAJB6 has been shown to interact with tau, the other major amyloid protein seen in AD. Downregulation of DNAJB6b increases the insoluble form of tau, while overexpression of DNAJB6b reduces tau aggregation. This suggests that DNAJB6b may also influence tau aggregation and the formation of neurofibrillary tangles (Chang et al., 2023). Additionally, when the HPD motif in the JD of DNAJB6 is mutated, interaction with Hsp70 is lost. However, this mutant does not lose its association with tau even though the suppression of aggregation is decreased, indicating that DNAJB6-Hsp70 interaction is important for decreased aggregation, but not for the ability of DNAJB6 to bind tau protein (Chang et al., 2023). The presence of DNAJB proteins in neurofibrillary tangles has been demonstrated, indicating its potential involvement in slowing the progression of AD (Cheetham et al., 1992).

In summary, research on DNAJB6 in various experimental models of neurodegeneration has highlighted its remarkable capacity to inhibit protein aggregation across a range of diseases. The protective role of DNAJB6 is now well established *in vitro*, in cellular models and animal models of HD and PD. The role of DNAJB6 in other neurodegenerative diseases is at present less well studied, but the findings so far suggest that DNAJB6 may play an equally important role in protecting against these diseases as well.

## Dysregulation of DNAJB6 in Clinical Cases of Neurodegenerative Diseases

The protein expression levels of DNAJB6 are dysregulated in multiple neurodegenerative diseases, suggesting its potential as a biomarker for disease progression and severity (Folke et al., 2021). In humans, the amount of total DNAJB6 is substantially increased in Lewy bodies, and DNAJB6 has been identified as a component in the core of the Lewy bodies in both the cortex and in the SN of PD patients (Durrenberger et al., 2009). Additionally, in a large gene expression screening, total *DNAJB6* mRNA was found to be dysregulated in SN of sporadic PD patients (Moran et al., 2006). In line with this, the human brain tissue expression of DNAJB6b was recently studied using an anti-DNAJB6b antibody (Folke et al., 2021). Total levels of DNAJB6b were found to be reduced in brain lysates from PD patients compared to controls, while total DNAJB6 levels were increased (Folke et al., 2021), consistent with results from Durrenberger et al. (2009). These findings suggest that the anti-aggregation activity of DNAJB6b may have clinical significance in neurodegenerative diseases, highlighting its potential as a therapeutic target.

In terms of non-neuronal cell types that express DNAJB6, it has been shown that DNAJB6 is expressed in astrocytes and is upregulated in astrocytes in the parkinsonian brain, suggesting a role for glial cells in PD (Durrenberger et al., 2009). However, the astrocytes in this study were not specifically labeled with a common astrocyte biomarker. In contrast, a recent clinical study employing multiple antibodies to label neurons, astrocytes, oligodendrocytes, and microglia specifically, revealed that DNAJB6 is almost exclusively present in neurons and oligodendrocytes of the human brain, and is sparsely present in other cell types such as astrocytes (2%–6% of astrocytes expressed DNAJB6) (Hentze et al., 2024). DNAJB6 expression may be increased in astrocytes of PD cases compared to non-PD cases, which could explain these differences. However, further research should be conducted to clarify these differences.

The study by Hentze et al. (2024) showed that DNAJB6 is abundantly expressed in oligodendrocytes, making it highly relevant to investigate whether DNAJB6 is an important protective factor in another synucleinopathy, namely multiple system atrophy (MSA). MSA is characterized by the accumulation of α-synuclein aggregates, in glial cytoplasmic inclusions (GCIs) within oligodendrocytes in the brain, which, interestingly, express α-synuclein at low levels in healthy brains (Asi et al., 2014). It has recently been shown that DNAJB6b is reduced in human brain MSA patient material (Folke et al., 2021), and therefore it would be interesting to explore whether DNAJB6 plays a role in suppressing α-synuclein in this disease as well. However, the α-synuclein aggregates formed as glial cytoplasmic inclusions in MSA (Jellinger, 2018) are different from the aggregates formed in PD. Therefore, further experiments should be conducted, to explore if DNAJB6 suppresses α-synuclein aggregation in MSA.

The expression of DNAJB6 has also been examined in clinical cases of progressive supranuclear palsy (PSP). PSP is a disorder characterized by the accumulation of hyperphosphorylated tau. Although research on DNAJB6 in PSP is less extensive, its role in tau aggregation suggests that it may contribute to the pathology of this disease as well (Chang et al., 2023). Recently, it was demonstrated that DNAJB6b is also downregulated in PSP brains (Folke et al., 2021). The presence of DNAJB6 in the context of tau pathology indicates its potential as a therapeutic target in PSP.

These clinical insights underscore the importance of understanding the functional mechanisms of DNAJB6 at a molecular level. Studies where DNAJB6 protein expression has been shown to be altered in human patient material are summarized in **Table 2**.

**Table 2:** DNAJB6 protein expression in clinical tissues

Disease	Dysregulation of DNAJB6 expression in clinical tissue	Cell type specific expression
PD	Durrenberger et al., 2009; n = 21	Durrenberger et al., 2009; neurons, astrocytes
	Folke et al., 2021; n = 13	
MSA	Folke et al., 2021; n = 13	Hentze et al., 2024; neurons, oligodendrocytes
PSP	Folke et al., 2021; n = 13	

MSA: Multiple system atrophy; PD: Parkinson’s disease; PSP: progressive supranuclear palsy.

## Structure and Oligomerization of DNAJB6

To explore the role of this chaperone in neuroprotection, a handful of studies have investigated the structure and oligomerization of DNAJB6, which has provided critical insights into how it binds amyloidogenic proteins. Structural studies of DNAJB6 protein are challenging due to its flexible regions and its tendency to form oligomers (Karamanos et al., 2019). As a result, our current understanding of the three-dimensional structure of DNAJB6 is limited. However, structural models of both monomeric and dimeric forms of DNAJB6b were made in 2018 by Söderberg et al. (2018). The dimer model suggested that the S/T-residues are solvent-accessible and form a peptide-binding cleft at the interface between the CTDs of the two subunits. This cleft may serve as the interaction site with the amyloid peptide, Aβ42. Furthermore, their analyses concluded that DNAJB6b oligomers have a varying number of subunits, ranging from 20 to 55, which presumably increases the number of available peptide interaction surfaces (Soderberg et al., 2018).

Subsequently, a solution structure of DNAJB6b lacking the S/T-rich region was solved using nuclear magnetic resonance by Karamanos et al. (2019). They found that removing the S/T-rich region perturbed oligomer formation, while keeping the monomeric structure intact (Karamanos et al., 2019), suggesting that the S/T-rich region plays a role in nucleating oligomerization. However, they discovered that DNAJB6b forms oligomers through its CTD, not the S/T-rich region, and the deletion of the 10 C-terminal amino acids unique to the DNAJB6b, further abolished oligomerization (Karamanos et al., 2019). This indicates that the oligomerization of DNAJB6a may differ, as DNAJB6a and DNAJB6b differ in their CTD. The role of the CTD has recently been investigated further, demonstrating that while the full-length DNAJB6b is inhibiting Aβ42-aggregation at both primary and secondary nucleation stages, the CTD alone primarily targets secondary nucleation (Osterlund et al., 2023).

Recently, a full-length three-dimensional model of DNAJB6b was generated, confirming that DNAJB6b is a highly dynamic protein (Adupa et al., 2024). These findings showed that DNAJB6b can adopt three distinct conformal states; closed, open, or extended, and that all three states exhibit an auto-inhibitory feature, suggesting that interaction with Hsp70 might require DNAJB6b to be substrate-loaded.

Furthermore, it has been shown, that a transition of the β1 strand (which includes S/T residues) from a twisted to a straight configuration leads to dimerization/oligomerization (Karamanos et al., 2020). These S/T residues are also part of a region crucial for the anti-aggregation activity of DNAJB6b (Kakkar et al., 2016b). Interestingly, a mutation in the middle of the β1 strand (T193A) of DNAJB6 may be linked to rare 2^nd^ hit cases of familial PD (Aslam et al., 2021). Cells expressing the T193A mutant DNAJB6 showed a slight increase in oligomerization (Cawood et al., 2022).

**Figure 4:**
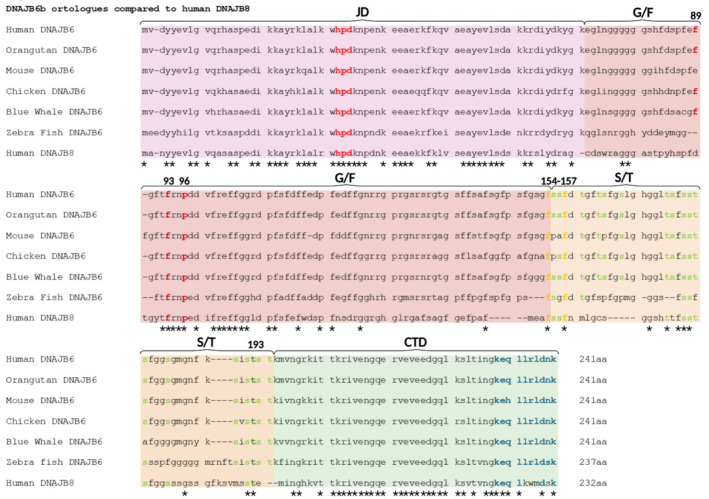
Sequence alignment of human DNAJB6b and selected orthologs. The four main domains/regions of the protein are marked with different colors. Important and/or functional amino acids are highlighted: red = HPD motif; dark red = mutations at these sites (F89I, F93L and P96R) have been associated with limb-girdle muscular dystrophy (LGMD) (Sarparanta et al., 2012; Thiruvalluvan et al., 2020); orange = mutations in the DNAJB8 protein at these sites (F148S and F151S, corresponding to F154 and F157 in DNAJB6) have been shown to be important for oligomerization of DNAJB8 (Ryder et al., 2024) light green = S and T residues of the S/T-rich region; dark green = mutation at this site (T193A) has been associated with Parkinson’s disease (Aslam et al., 2021); blue = last ten amino acids unique to DNAJB6b isoform compared to DNAJB6a isoform. *Denotes where the specific amino acid is conserved across all sequences aligned. Created with BioRender.com. DNAJB: DNAJ homolog subfamily B; G/F: glycine/phenylalanine-rich region; JD: J-domain; S/T: serine/threonine-rich region.

Additional insights into the role of oligomerization primarily come from studies of DNAJB8, a protein closely related to DNAJB6, in sequence and function. In a recent study, Ryder et al. (2024) demonstrated that DNAJB8 variants unable to oligomerize, due to introduced point mutations, were more specific in binding to misfolded tau seeds, while they retained capacity to reduce protein aggregation *in vitro* and in cells. They found that amino acids F148 and F151 were particularly important for DNAJB8 oligomerization as they were crucial for the structure of a “zipper motif” (Ryder et al., 2024). Interestingly, this zipper motif is partly conserved in DNAJB6 as well (**Figure 4**).

Consistent with these findings, a study by Carlsson et al. (2024) showed that large oligomers of DNAJB6 are essentially inactive in terms of anti-aggregation activity, and to effectively inhibit amyloid aggregation, these large oligomers must dissociate into smaller subunits. These data indicate that even though DNAJB6b has a strong propensity to oligomerize, it is the smaller assemblies rather than larger oligomers that are important for the anti-aggregation activity of this chaperone. Another study suggests that oligomer formation may be concentration-dependent, indicating that *in vitro* studies using high concentrations of DNAJB6b might not accurately represent its assembly state under physiological conditions (Carlsson et al., 2023). It is possible that chaperone activity, under normal biological conditions, occurs in a smaller assembly state.

Interestingly, a related study by Velasco-Carneros et al. (2023) suggests that the oligomers of DNAJA2 may function as a storage state from which the protein disassembles to engage with client proteins during conditions of protein misfolding stress, which may also be the case for larger DNAJB6b oligomeric assemblies. This idea supports the notion that controlled oligomerization and dissociation play crucial roles in the chaperone activity of these proteins during stress responses.

In conclusion, although the full structure of DNAJB6 has yet to be resolved, it is evident that both the CTD and the S/T-rich region contribute to oligomer formation. As the S/T-rich region in particular appears to influence both client interaction and oligomer formation, and the activity of DNAJB6b is thought to rely on its assembly state, this region may be important both for regulating oligomerization and determining when DNAJB6 can interact with substrates.

## Functional Roles of DNAJB6

One of the most characteristic functions of DNAJB6 is its role as a co-chaperone for Hsp70, which in turn is critical for its ability to prevent aggregation of some amyloidogenic proteins. Initially, Chuang et al. (2002) confirmed *in vitro* that DNAJB6b acts as an effective co-chaperone of Hsp70, showing that DNAJB6b increases the ATP hydrolysis rate by approximately eight-fold compared to Hsp70 alone.

Besides its critical role in preventing amyloid protein aggregation, DNAJB6 has many other functions in the cell and specific tissues. For instance, DNAJB6 plays a critical role in placental development (Hunter et al., 1999) and neurulation in embryogenesis (Watson et al., 2009). Additionally, DNAJB6b has been shown to co-localize with K18 keratin filaments, and specifically interact with the coil II region of K18 through its CTD, suggesting that it helps regulate the organization of the cytoskeleton (Izawa et al., 2000). This was further supported by findings that DNAJB6b mutant trophoblast cells exhibited a collapsed keratin network and contained large keratin inclusion bodies, highlighting the importance of DNAJB6b in maintaining the turnover of intermediate filaments (Watson et al., 2007). The failure of chorioallantoic attachment in DNAJB6b KO mice (Watson et al., 2007), as well as its role in placental development, may therefore be related to its function in keratin organization. Whereas DNAJB6b has been associated with keratin filaments, recent research has demonstrated a role for DNAJB6a in microtubule organization in mitosis (Rosas-Salvans et al., 2018, 2019). These functions highlight crucial role of DNAJB6 in embryonic development, as well as supporting cellular stability and overall cell structure and dynamics.

Interestingly, Desai et al. (2024) recently showed that DNAJB6 KO *D. melanogaster* has impaired long-term memory and that DNAJB6 regulates oligomerization of the protein ortholog of CBEP in humans (orb2), which is important for long-term memory formation. KO of DNAJB6 is not possible in mammalian organisms, which is most likely due to its crucial role in placenta development (Hunter et al., 1999). However, a mammalian model with conditional KOs of *DNAJB6* gene in the brain would make it possible to explore the role of DNAJB6 in memory as well as it could be explored if KO of DNAJB6 accelerates amyloid protein aggregation in the brain, which cellular studies suggest that it would (Aprile et al., 2017; Rodriguez-Gonzalez et al., 2020).

Additionally, DNAJB6 plays a role in the regulation of the cell cycle. It has been shown to promote cell cycle arrest via promoting the nuclear import of Schlafen1 (Zhang et al., 2008). In this regard, it is interesting to note that the expression of DNAJB6 is dependent on the cell cycle stage (Dey et al., 2009), and that DNAJB6 is involved in NPC biogenesis during interphase (Kuiper et al., 2022). Therefore, it is perhaps not surprising that DNAJB6 may have a protective role in cancer. DNAJB6a reduces Akt signaling and cyclin E1 expression, thereby inhibiting the cell cycle, which has been shown to be Hsp70 dependent. Moreover, patients exhibiting nuclear expression of DNAJB6 in esophageal cancer had a better survival prognosis compared to controls (Yu et al., 2015). In addition, it was demonstrated that expression of DNAJB6 is lost in advanced breast cancer, and overexpression in cancer cells inhibited tumor growth in a nude mouse model (Mitra et al., 2008). DNAJB6 inhibits the Wnt/β-catenin pathway, which provides a possible explanation for its anti-tumorigenic effects (Mitra et al., 2010).

Whereas DNAJB6 expression plays a protective role against toxic amyloidogenic protein aggregation in the brain and seemingly also in cancer, there are dominant mutations in the gene encoding DNAJB6 that cause limb-girdle muscular dystrophy (LGMD), which leads to muscle weakness and atrophy (D’Este et al., 2025). Several studies have identified missense mutations in the *DNAJB6* gene that leads to LGMD (Harms et al., 2012; Couthouis et al., 2014). The mutations in the *DNAJB6* gene that cause LGMD mainly result in missense mutations in the protein at residues Phe89, Phe93, and Pro96 in the G/F-rich region, which are highly conserved residues of DNAJB6 (**Figure 4**). However, it is still not fully understood what the functional effects of these dominant mutations are and why they only cause cell death in skeletal muscle cells. Future findings on what functional consequences these mutations have for DNAJB6 may shed important light on how DNAJB6 works tissue specifically and in general. Multiple additional mutations in DNAJB6 have also been found to cause LGMD, which has been reviewed more comprehensively elsewhere (Ruggieri et al., 2016; Sarparanta et al., 2020).

## Therapeutic Perspectives of Targeting DNAJB6

Here we have reviewed research, which shows that DNAJB6 protects against amyloid protein aggregation in multiple diseases. One of the well-studied mechanisms is when DNAJB6 inhibits the early stages of amyloid protein aggregation in models of HD (Mansson et al., 2014a; Kakkar et al., 2016b). This mechanism presents a targeted approach whereby toxic amyloid protein aggregation can be prevented in this monogenetically inherited disease, caused by CAG extensions in the *Huntingtin* gene (Saade and Mestre, 2024). DNAJB6 also inhibits aggregation of the protein α-synuclein (Aprile et al., 2017) in what may be a similar manner. Moreover, DNAJB6 also ships misfolded amyloid proteins for degradation causing a lowering of the protein levels causing the formation of toxic aggregates (Deshayes et al., 2019). The cause of PD is multifactorial, but α-synuclein aggregation is believed to be a driving force, as it is found mutated in some cases of inherited PD (Polymeropoulos et al., 1997), as well as it is the most abundant protein in the Lewy bodies, where it is primarily found in an aggregated form (Spillantini et al., 1997). Therefore, the possibility of inhibiting α-synuclein aggregation by increasing DNAJB6 activity could also be a promising therapeutic goal in PD.

As DNAJB6 inhibits the early stages of amyloid protein aggregation, it is possible that it may not be effective in reversing later phases in neurodegenerative diseases. However, it is plausible that even at late disease stages there is still *de novo* aggregation occurring and that DNAJB6 may thereby delay disease progression. Therefore, enhancing DNAJB6 expression or activity could still hold great therapeutic promise.

One potential approach to deliver DNAJB6 to target cells might be through the use of EVs. EVs are naturally occurring lipid bilayer structures that can encapsulate proteins, lipids, and nucleic acids, facilitating intercellular communication and cargo delivery (Visnovitz, 2024). EVs enriched with DNAJB6 have been proposed as a novel strategy for delivering chaperone activity to cells (Joshi et al., 2021, 2022). A previous study has shown that EVs loaded with DNAJB6 can effectively suppress polyQ aggregation in both *in vitro* and *in vivo* models of HD (Joshi et al., 2022), indicating their potential as a therapeutic vehicle. The encapsulation of DNAJB6 within EVs not only protects the chaperone from degradation but also enhances its bioavailability and efficacy in target cells, potentially restoring protein homeostasis in neurodegenerative contexts (Akyuz et al., 2024). Moreover, the natural origin and low immunogenicity of EVs make them particularly attractive for therapeutic applications, especially in neurodegenerative diseases where traditional delivery methods may be less effective. It is however crucial to consider DNAJB6’s concentration-dependent self-association into micellar or oligomeric structures (Carlsson et al., 2023). Notably, these oligomeric forms exhibit reduced efficiency in preventing amyloid protein aggregation (Carlsson et al., 2024). Therefore, increasing DNAJB6 levels, either by delivery or upregulation, may not necessarily enhance its protective function.

There is clinical evidence that the levels of DNAJB6 are dysregulated in multiple neurodegenerative diseases, which adds to the hypothesis that DNAJB6 may play a protective role in these diseases (Folke et al., 2021). The ability of DNAJB6 to modulate the aggregation of various misfolded proteins suggests that it may serve as a universal chaperone for multiple neurodegenerative diseases, making it a versatile target for therapeutic interventions. Future therapeutic strategies could focus on enhancing DNAJB6 expression and/or activity through small molecules or compounds. Compounds that upregulate chaperone expression or that enhance or mimic their activity could potentially be used in conjunction with existing treatments to improve outcomes for patients with neurodegenerative diseases. Screening for drugs that enhance the formation of DNAJB6 dimers may be a promising therapeutic strategy as dimeric form of DNAJB6 is crucial for its anti-aggregation activities (Soderberg et al., 2018).

## Conclusions and Perspectives

In summary, DNAJB6 has emerged as a key player in the field of neurodegenerative diseases, owing to its ability to inhibit the aggregation of multiple amyloidogenic proteins that are pathological hallmarks of these disorders. Through studies spanning from *in vitro*, cellular and animal models to human tissues, DNAJB6 has been shown to be a critical chaperone with therapeutic potential against the buildup of pathological protein species, such as polyQ, α-synuclein, and other amyloid aggregates. Its two isoforms add further complexity to its functions, with each isoform contributing to distinct roles in cellular protein homeostasis.

**Figure 5:**
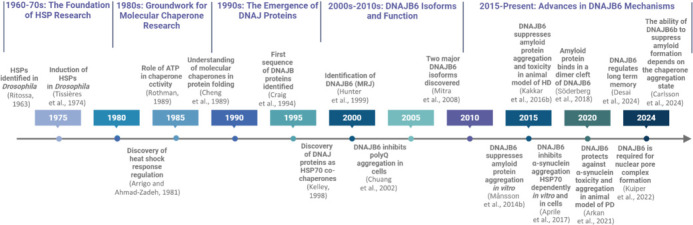
Timeline of key events in the discovery and characterization of DNAJ homolog subfamily B (DNAJB) proteins. This timeline illustrates the significant milestones in the research of DNAJB proteins, particularly DNAJB6 and its isoforms (DNAJB6a and DNAJB6b), from the initial identification of HSP in the 1970s to recent advances in understanding their roles in cellular processes and diseases. Each event is marked along the timeline, highlighting the progression of research and the evolving understanding of these critical molecular chaperones. Created with BioRender.com. HSP: Heat shock protein.

A timeline of key events in the discovery and characterization of DNAJB proteins highlights the understanding of their molecular mechanisms. From the identification of HSPs to the involvement of JDPs in neuroprotection, the multifaceted roles of DNAJB6 are unraveled (**Figure 5**). Despite recent and critical advances, there are still unanswered questions. For instance, we do not know if the lack of DNAJB6 expression in the brain of mammalian animal models of neurodegenerative diseases such as HD or PD will accelerate disease progression. Although the accumulated research so far indicates that the dimer is the form of DNAJB6b that inhibits the amyloid protein aggregation, we do not know whether slightly higher order oligomers also do and we do not know specifically how large these oligomers can be before they no longer inhibit amyloid protein aggregation, and at that stage might be in some higher order oligomer storage form of the protein. While DNAJB6b is known to inhibit amyloid protein aggregation, it is unclear whether DNAJB6a can also do so in the absence of DNAJB6b. No studies have specifically knocked out the DNAJB6b isoform, nor has the effect of recombinant DNAJB6a on amyloid protein aggregation been investigated. In summary, we believe DNAJB6 is a molecule well worthwhile studying further to understand cellular mechanisms that prevent neurodegenerative disease progression, and by combining with drug screenings, this could in turn lead to novel avenues of treating these diseases, for which there are at present very few long-lasting treatment options.

## Data Availability

*Not applicable.*.
